# Distinct patterns and prognostic values of tumor-infiltrating macrophages in hepatocellular carcinoma and gastric cancer

**DOI:** 10.1186/s12967-017-1139-2

**Published:** 2017-02-15

**Authors:** Jin-Qing Li, Xing-Juan Yu, Yong-Chun Wang, Li-Yun Huang, Chao-Qun Liu, Limin Zheng, Yu-jing Fang, Jing Xu

**Affiliations:** 1 Collaborative Innovation Center of Cancer Medicine, State Key Laboratory of Oncology in South China, Sun Yat-sen University Cancer Center, Guangzhou, 510060 People’s Republic of China; 2 Department of Pathology, Sun Yat-sen University Cancer Center, Guangzhou, 510060 People’s Republic of China; 3 School of Life Sciences, Sun Yat-sen University, Guangzhou, 510060 People’s Republic of China

**Keywords:** Macrophage, CD204, CD169, Prognosis, Hepatocellular carcinoma, Gastric cancer

## Abstract

**Background:**

Macrophages (Mφs) constitute a major component of the leukocyte infiltrate and perform distinct roles in different tumor microenvironments. This study aimed to characterize the distribution, composition and prognostic value of Mφs in hepatocellular carcinoma (HCC) and gastric cancer (GC).

**Methods:**

Immunohistochemistry and immunofluorescence were used to identify Mφ subsets in HCC and GC tissues. Kaplan–Meier analysis and Cox regression models were applied to estimate the overall survival (OS) for HCC and GC patients.

**Results:**

The results showed that the density of Mφs decreased in the intra-tumor region (IT) of HCC, but remarkably increased in the IT of GC, as compared with their non-tumor regions (NT). In HCC, most CD68^+^ Mφs were CD204^+^ and CD169^+^ cells in the NT region; however, there was a significant decrease in the percentage of CD169^+^ Mφ in the IT region. In contrast, CD68^+^ Mφs comprised a smaller percentage of CD204^+^ than the CD169^+^ subpopulation in the NT region, while more CD204^+^ but fewer CD169^+^ cells were present in the IT region of GC. The density of CD204^+^ Mφs correlated with poor prognosis in HCC, and CD169^+^ Mφs were associated with good survival in both HCC and GC. Moreover, the combination of low numbers of CD204^+^ and high numbers of CD169^+^ Mφs was associated with improved OS in both GC and HCC.

**Conclusions:**

Mφs display tissue-specific distributions and distinct composition patterns in HCC and GC tissues. Our results suggested that different types of tumors might use diverse strategies to reconstitute Mφ patterns to promote tumor progression.

**Electronic supplementary material:**

The online version of this article (doi:10.1186/s12967-017-1139-2) contains supplementary material, which is available to authorized users.

## Background

Macrophages (Mφs) are essential components of the innate immune system and are widely distributed throughout the body [1]. High numbers of tumor-associated Mφs are found in tumors and constitute a major component of the inflammatory infiltrate in virtually all malignancies [2, 3]. The variety of local tumor environmental conditions could shape the Mφ identity and Mφs have both pro- and anti-tumorigenic functions, thus making them an attractive target for novel anti-cancer therapies [4, 5].

Hepatocellular carcinoma (HCC) and gastric cancer (GC) are the most common malignancies and leading causes of cancer mortality worldwide [6]. The increasing incidence of HCC has been attributed to the dissemination of hepatitis B (HBV) and hepatitis C (HCV) virus infection; while *Helicobacter pylori* infection is the principle risk factor for the development of the chronic gastric inflammation that progresses to GC [7–9]. Despite these different pathogeneses, emerging data suggest that tissue-specific functions could also determine the source and function of Mφs [10–12]. In the gastrointestinal system, Mφs are derived from circulating monocytes and function as sentinels of the immune system to avoid collateral damage by secretion of the pro-inflammatory cytokines that are induced by bacterial products [13]. By contrast, in the liver, Mφs are predominantly self-renewed from resident stem cells that originated from the fetal yolk-sack during homeostasis, but can also be recruited from blood monocytes after liver injury [14]. The distinct local environments and cell sources might contribute to the development of Mφs in these two types of tumor; however, presently there is a lack of human studies comparing the distribution, phenotype and clinical relevance of Mφs in these tumors.

Diverse Mφ subpopulations can be distinguished based on the expression of several specific markers. CD68, a pan-Mφ marker, has been used widely to evaluate Mφ density in different types of tumors. Our and other groups have shown that a high density of CD68^+^ Mφs correlates with a negative outcome in HCC patients; however, conflicting data were produced in GC [15–18]. To potentially represent more selective Mφs, some other phenotypic markers of Mφs have been reported. Biomarkers such as CD163, CD204 which are considered to be associated with M2 activation state, have been found to correlate with negative outcomes in multiple tumor types [19–24]. CD204 is a phagocytic pattern-recognition receptor that is primarily expressed on myeloid lineage cells. The high density of CD204^+^ Mφs have been reported to be associated with poor outcomes in both GC and HCC patients [25, 26]. Mφ could also possess anti-tumor phenotypic state (M1), which were correlated with good prognosis in some tumors [27]. Our recent study demonstrated that CD169^+^ Mφs can dominate anti-tumor immunity and are correlated with improved prognosis in HCC patients [28]. However, there is a lack of studies examining the differences and similarities in the composition pattern of Mφs subtypes in different types of tumors.

In this study, we assessed the tissue-specific distribution and composition of different Mφ subpopulations in HCC and GC tissues, and investigated the prognostic significance of these Mφs in samples from 188 HCC and 138 GC patients.

## Methods

### Patients and specimens

Archived, formalin-fixed, paraffin-embedded (FFPE) tissues from 188 HCC patients and 138 GC patients who had all undergone radical resection for tumors at the Sun Yat-Sen University Cancer Center between 2002 and 2012 were enrolled in this study. Patients who exhibited signs of distant metastasis and had received anti-cancer therapies before surgery, or experienced concurrent autoimmune disease, were excluded. The diagnosis of HCC and GC in each patient was confirmed histopathologically. The tumor stage was determined according to the tumor-node-metastasis (TNM) classification system of the International Union Against Cancer, 7th Edition. Data was censored at the last follow-up for surviving patients. Overall survival (OS) was defined as the interval between the time of surgery and either the last follow-up or death.

This study conformed strictly to the ethical guidelines of the Declaration of Helsinki and was approved by the Research Ethics Committee of Sun Yat-Sen University Cancer Center. Written informed consent was obtained from all patients before sample collection. All samples were coded and data was stored anonymously. The clinicopathological characteristics of the patients are summarized in Table 1.

**Table 1:** Clinicopathological characteristics of the patients

Variables	No. and (%)
*HCC patients*	
No. of patients	188
Age (median; range), years	50; 13–76
Gender (male/female)	159/29 (84.6/15.4)
HBV infection (no/yes)	19/169 (10.1/89.9)
Alpha-fetoprotein, ng/mL (≤2 5/>25)	74/114 (39.4/60.6)
Child–Pugh class (A/B)	175/13 (93.1/6.9)
Tumor number (single/multiple)	144/44 (76.6/23.4)
Tumor size, cm (≤5/>5)	80/108 (42.6/57.4)
Vascular invasion (absent/present)	177/11 (94.1/5.9)
TNM stage (I/II/III)	130/16/39 (69.1/10.1/20.8)
Histological grade (I/II/III/other)	125/63 (66.5/33.5)
*GC patients*	
No. of patients	138
Age (median; range), years	69; 28–78
Gender (male/female)	100/38 (72.5/27.5)
Tumor size, cm (≤4/>4)	46/92 (33.3/66.7)
Tumor depth (pT1/pT2/pT3/pT4)	3/10/34/91 (2.2/7.2/24.7/65.9)
Lymph node metastasis (pN0/pN1/pN2/pN3)	29/31/27/51 (21.0/22.5/19.5/37.0)
TNM stage (IA/IB/II/IIIA/IIIB/IIIC)	3/6/5/25/32/32/35 (2.2/4.3/3.6/18.1/23.2/23.2/25.4)
Histological grade (I/II/III/other)	3/32/90/11 (2.2/23.2/65.2/8.0)

*HCC* hepatocellular carcinoma, *GC* gastric cancer, *HBV* hepatitis B virus, *TNM* tumor-lymph node-metastasis

### Immunohistochemistry (IHC) and immunofluorescence staining

IHC was performed using a two-step method (DakoCytomation, Glostrup, Denmark) using protocols described in our previous studies [29, 30]. Sections of FFPE tissues were cut using a microtome, and then sequentially dried, dewaxed, and re-hydrated with xylene and a decreasing ethanol series. Endogenous peroxidase activity was then blocked with 0.3% H_2_O_2_ for 10 min. For antigen retrieval, sections were steamed in 10 mM citrate buffer (pH 6.0) for 10 min. Glass slides were incubated overnight at 4 °C with anti-CD204 (Transgenic, Kumamoto, Japan), anti-CD169 (R&D Systems, Minneapolis, MN, USA), or anti-CD68 (DakoCytomation, Carpinteria, CA, USA) antibodies. Horseradish peroxidase-conjugated anti-rabbit and anti-mouse antibodies from Dako EnVision systems (DakoCytomation) were used as secondary detection reagents and the immunoreactivities were visualized using 3,3′-diaminobenzidine (DAB). All sections were lightly counterstained with Mayer’s Hematoxylin Solution (Sigma) and mounted using non-aqueous Permount™ mounting medium. Negative controls comprised slides for which the primary antibodies were replaced by the same concentration of an irrelevant, isotype-matched antibody.

Double immunofluorescent staining was carried out as previously described [30]. Briefly, re-hydrated FFPE sections were incubated at 4 °C overnight with mouse anti-human CD68, rabbit anti-human CD204, or sheep anti-human CD169 antibodies. The sections were then incubated for 30 min at 37 °C with a mixture of primary-antibody-matched fluorescently labeled secondary antibodies (Invitrogen; Carlsbad, CA, USA). Nuclei were counterstained using 4′,6-diamidino-2-phenylindole (DAPI).

### Image quantification

To quantify CD204^+^ and CD169^+^ cell density, the Vectra-Inform image analysis system (Perkin-Elmer/Applied Biosystems, Foster City, CA, USA) was used, as described in a previous study [28, 30]. Target signals were quantified in selected tissues and cellular compartments of interest. The percentage of each immune cell subset was calculated by dividing the absolute number of each cell subset by area of the tissue surface.

Quantification methods for immunofluorescence were performed as previously described [30]. Immunofluorescence images were captured using a confocal microscope (Olympus, Essex, UK) and analyzed using FV10-ASW Viewer (Olympus, Essex, UK). The number of single-positive or double-positive cells in each of five representative fields at 400× magnification were counted. From these numbers, the proportions of CD204^+^ or CD169^+^ cells in CD68^+^ Mφs were calculated as: (number of CD204^+^CD68^+^ cells)/(number of CD68^+^ Mφs), or (number of CD169^+^CD68^+^ cells)/(number of CD68^+^ Mφs).

### Statistical analyses

OS curves were obtained using the Kaplan–Meier method, and compared using the log-rank test for each prognostic variable. Variables with effects on survival in univariate analysis were included in a multivariate Cox proportional hazard regression model, which was used to estimate the adjusted hazard ratio (HR) and 95% confidence interval (CI), and to identify independent prognostic factors. Subgroups of each immunostaining parameter were divided by the median values. Associations between immunostaining parameters and clinicopathological features were evaluated using the *χ* ^2^ test or Fisher’s exact test, as appropriate. A threshold of *P* < 0.05 denoted statistical significance. SPSS 20.0 (IBM) was used for the statistical analyses.

## Results

### Distribution of Mφs in HCC and GC

**Fig. 1:**
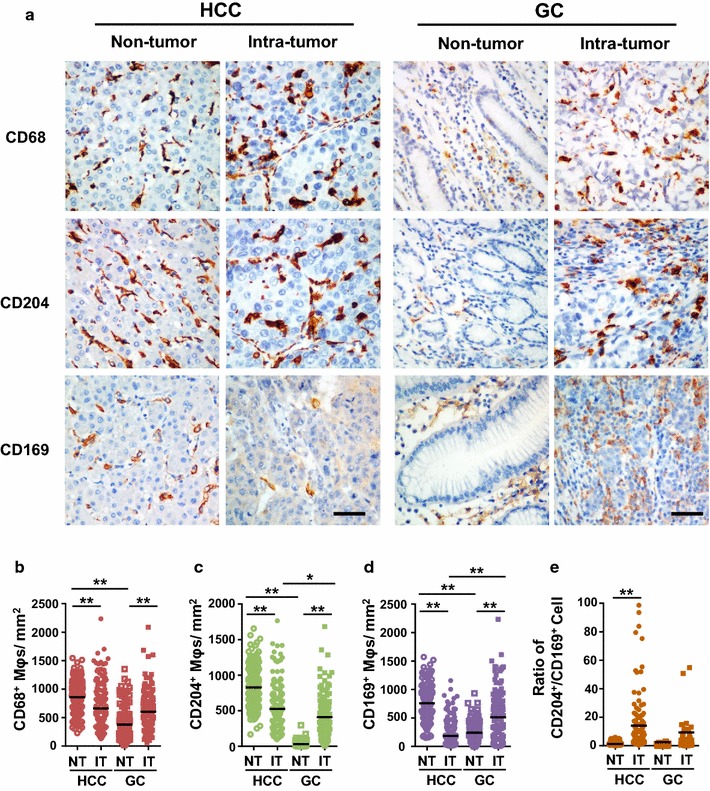
Mφs distributions in the non-tumor (NT) and intra-tumor (IT) regions of hepatocellular carcinoma (HCC) and gastric cancer (GC). **a** Representative immunohistochemistry images of CD68^+^ Mφs, CD204^+^ Mφs, and CD169^+^ Mφs in human HCC and GC tissues. *Scale bar*, 100 μm. **b**–**d** The numbers of CD68^+^ Mφs (**b**), CD204^+^ Mφs (**c**), CD169^+^ Mφs (**d**) and CD204^+^/CD169^+^ Mφs ratios (**e**) in the NT and IT regions of human HCC and GC tissues. Cell numbers were calculated as the cell count per ×400 field. Data are expressed as mean ± SEM. *P < 0.05; **P < 0.01

To evaluate the in situ distribution of different Mφ subpopulations, we used immunostaining to detect CD68^+^ Mφs, CD204^+^ Mφs, and CD169^+^ Mφs in the non-tumor (NT) and intra-tumor (IT) areas of HCC and GC. Clear and distinguishable staining was observed for all the phenotypic markers. In HCC, Mφs were evenly distributed in the parenchyma of both the NT and IT regions. In GC, Mφs were gathered in the stromal area surrounded the glandular tubes of gut tissue, but were scattered distributed in the tumor nest (Fig. 1a).

We compared the density of Mφs in the NT and IT regions of HCC and GC. Statistics showed that the numbers of CD68^+^ Mφs were high in the NT of HCC, but were relatively low in the NT of GC tissues, with mean (±SEM) densities of 859 ± 19, and 378 ± 28 in HCC and GC, respectively (*P* < 0.001; Fig. 1b). However, the density decreased in the IT of HCC (660 ± 28), while it remarkably increased in the IT of GC (604 ± 29). We also compared the distribution of different Mφ subpopulations. In HCC, the densities of both CD204^+^ Mφs (826 ± 77 and 526 ± 23 in NT and IT, respectively; *P* < 0.001; Fig. 1c) and CD169^+^ Mφs (760 ± 22 and 187 ± 16 in NT and IT, respectively; *P* < 0.001; Fig. 1d) also decreased in the IT compared with the NT region. In GC, only a few CD204^+^ Mφs were found in the NT, but they were significantly enriched in the IT (31 ± 5 and 411 ± 27 in the NT and IT, respectively; *P* < 0.001; Fig. 1c). Moreover, CD169^+^ Mφs could be detected in the NT region and were also increased in the IT of GC (242 ± 20 and 514 ± 37 in the NT and IT, respectively; *P* < 0.001; Fig. 1d). In addition, the ratios of CD204^+^/CD169^+^ Mφs were significantly increased in IT as compared with NT regions of HCC but not in GC (1.2 ± 0.05 and 14.1 ± 1.9 in the NT and IT, respectively; *P* < 0.01; Fig. 1e). Taken together, the distribution of Mφs in the NT and IT areas differed in HCC and GC.

### Composition patterns of Mφs in HCC and GC

**Fig. 2:**
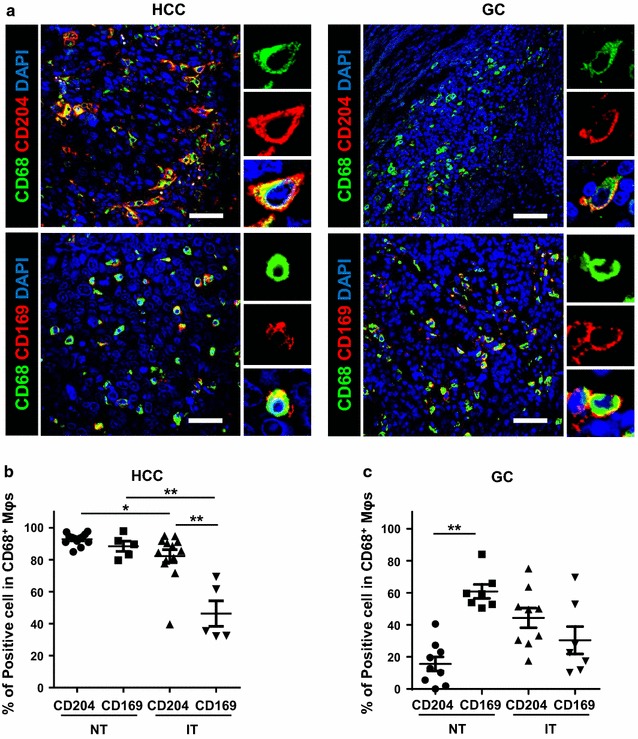
Composition patterns of Mφs subpopulations in CD68^+^ Mφs of HCC and GC intra-tumor tissues. **a** Paraffin-embedded tissue sections (n = 5) were subjected to three-color immunofluorescence for CD204 (*red*) or CD169 (*red*) with CD68 (*green*) and DAPI counterstaining (*blue*) in the intra-tumor regions of HCC and GC. **b**–**c** Percentage of CD204^+^ Mφs and CD169^+^ Mφs subpopulations in CD68^+^ Mφs of HCC (**b**) and GC (**c**). Data are expressed as mean ± SEM. *P < 0.05; **P < 0.01

CD68 is always used as a pan-Mφ marker, while CD204 and CD169 might represent different Mφ subpopulations with pro- or anti-tumor functions during tumor progression. Multiple immunofluorescence staining and confocal analyses confirmed that most CD204^+^ and CD169^+^ cells are CD68^+^ Mφs in both the NT and IT areas of HCC and GC (Fig. 2a; Additional file 1: Figures S1 and S2).

We then examined the proportion of each Mφ subpopulation within CD68^+^ Mφs in HCC and GC. As shown in Fig. 2b, most CD68^+^ Mφs are CD204^+^ (92.8 ± 1.0%) and CD169^+^ (88.4 ± 3.2%) cells in the NT area of HCC; however, the phenotype changed in the IT area, as shown by a significant decrease in the percentage of CD169^+^ Mφs (*P* = 0.004; Fig. 2b). Moreover, the CD169^+^ (46.3 ± 8.0%) proportion was significantly lower than that of the CD204^+^ (82.3 ± 4.1%; *P* < 0.001; Fig. 2b) subpopulation within total CD68^+^ Mφs in the IT region of HCC.

The composition of Mφs displayed a different pattern in GC, in that CD68^+^ Mφs comprised fewer CD204^+^ cell (15.7 ± 4.4%) than CD169^+^ cell (60.9 ± 4.3%) in the NT region (*P* < 0.001; Fig. 2c). In contrast, more CD204^+^ (44.3 ± 6.1%) but fewer CD169^+^ (30.4 ± 8.5%) cells were found in CD68^+^ Mφs in the IT region. Notably, the CD169^+^ subpopulation of Mφs was also significantly decreased in the IT compared with the NT region of GC (*P* = 0.008; Fig. 2c).

### Prognostic roles of CD204^+^ and CD169^+^ Mφ in HCC and GC

**Fig. 3:**
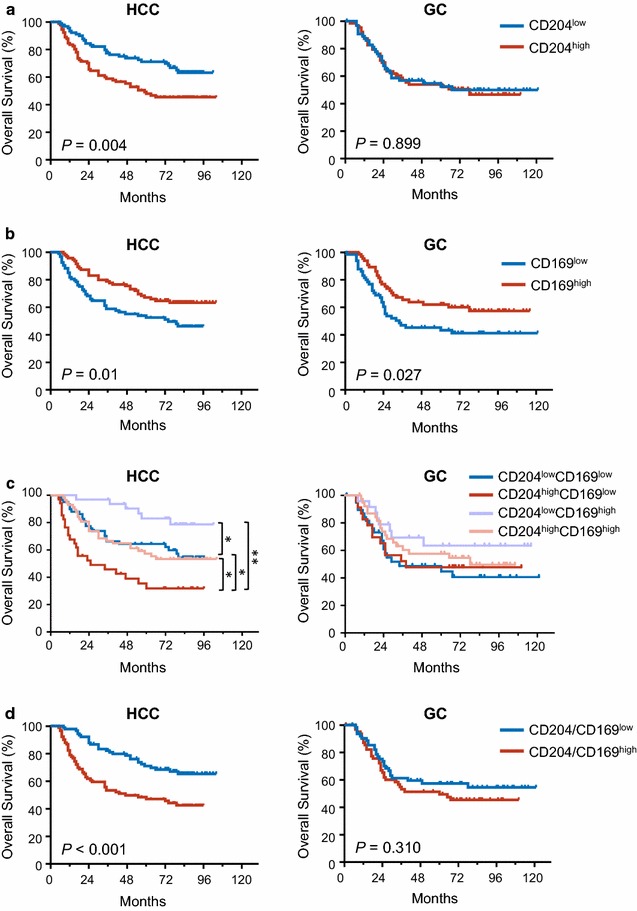
Cumulative overall survival curves of CD204^+^ Mφs and CD169^+^ Mφs for HCC and GC patients. Overall survival was estimated using the Kaplan–Meier method and compared using the log-rank test for CD204^+^ Mφs (**a**), CD169^+^ Mφs (**b**), the Mφ index (**c**) and Mφ ratio (**d**) in HCC and GC patients. *P < 0.05; **P < 0.01

The relationship between the densities of CD204^+^/CD169^+^ Mφs and patient survival was further investigated. Patients were divided into two groups, based on the median value of CD204^+^ or CD169^+^ Mφ density in the IT regions of HCC (median density, 460 and 112 for CD204^+^ and CD169^+^ Mφ, respectively) and GC (median density, 352 and 452 for CD204^+^ and CD169^+^ Mφ, respectively). Kaplan–Meier survival analysis revealed a negative correlation between the density of CD204^+^ Mφs and the OS of HCC patients (*P* = 0.004; Fig. 3a); however, no significant association was found for GC patients (*P* = 0.899; Fig. 3a). In contrast, a high density of intra-tumor CD169^+^ Mφs predicted favorable survival in both HCC (*P* = 0.01) and GC (*P* = 0.027; Fig. 3b) patients (Additional files 2, 3).

To further assess whether CD204^+^ or CD169^+^ Mφ density could be used as an independent predictor of OS, we performed multivariate Cox proportional hazards analysis. As shown in Table 2, the CD169^+^ Mφ density was associated with a decreased risk of death in HCC (HR 0.561, 95% CI 0.358–0.878, *P* = 0.011) and GC (HR 0.569, 95% CI 0.343–0.943, *P* = 0.029) patients. By contrast, the CD204^+^ Mφ density was associated with an increased risk of death in HCC (HR 1.922, 95% CI 1.217–3.034, *P* = 0.005), but no significant association was found for GC patients (HR 1.033, 95% CI 0.625–1.709, *P* = 0.899). Clinicopathological variables that were shown to be significant in the univariate analysis were used as covariates in the multivariate analysis. We found that the CD169^+^ Mφ density could act as an independent prognostic factor for OS in both HCC (HR 0.436, 95% CI 0.270–0.703, *P* = 0.001) and GC (HR 0.587, 95% CI 0.354–0.974, *P* = 0.039) patients.

**Table 2:** Univariate and multivariate analyses of variables associated with overall survival

Variables	Univariate			Multivariate		
	HR	95% CI	*P* ^a^	HR	95% CI	*P* ^a^
HCC patients						
Gender (male/female)	1.333	0.747–2.377	0.331			
HBV infection (no/yes)	0.863	0.431–1.729	0.678			
Alpha-fetoprotein, ng/mL (≤25/>25)	1.023	1.003–1.043	*0.027*			NA
Child–Pugh class (A/B)	1.626	0.782–3.378	0.193			
Histological grage (I/II/III/other)	1.42	0.901–2.237	0.131			
Tumor number (single/multiple)	3.178	2.008–5.031	< *0.0001*			NA
Tumor size, cm (≤5/>5)	1.838	1.154–2.927	*0.01*	1.614	0.997–2.613	0.051
Vascular invasion (absent/present)	3.832	1.825–8.046	< *0.0001*	2.667	1.208–5.888	*0.015*
TNM stage (I vs. II + III)	3.383	2.164–5.289	< *0.0001*	2.838	1.765–4.564	*0.0002*
${\text{CD2}}0 4_{IT}^{ + }$ cells (low/high)	1.922	1.217–3.034	*0.005*	2.125	1.298–3.478	*0.003*
${\text{CD169}}_{IT}^{ + }$ cells (low/high)	0.561	0.358–0.878	*0.011*	0.436	0.270–0.703	*0.001*
GC patients						
Gender (male/female)	1.189	0.702–2.013	0.52			
Tumor size, cm (≤4/>4)	1.655	0.951–2.881	0.075			
Tumor depth (pT1 + pT2 + pT3/pT4)	1.251	0.744–2.105	0.398			
Lymph node metastasis (pN0 + pN1/pN2 + pN3)	1.999	1.192–3.353	*0.009*	2.012	1.178–3.437	*0.011*
TNM stage (I + II vs. III + IV)	2.031	1.103–3.738	*0.023*			NA
Histological grage (I/II/III/other)	1.18	0.772–1.804	0.445			
${\text{CD2}}0 4_{IT}^{ + }$ cells (low/high)	1.033	0.625–1.709	0.899			
${\text{CD169}}_{IT}^{ + }$ cells (low/high)	0.569	0.343–0.943	*0.029*	0.587	0.354–0.974	*0.039*

Cox proportional hazards regression model; variables that were associated with overall survival in the univariate analysis were adopted as covariates in the multivariate analysis and were entered into the equation using the forward likelihood ratio method

*HCC* hepatocellular carcinoma, *GC* gastric cancer, *HBV* hepatitis B virus, *TNM* tumor-lymph node-metastasis, *CI* confidence interval, *NA* not applicable, *IT* intra-tumor

^a^Italic values indicate significance of *p* value (*p* < 0.05)

**Fig. 4:**
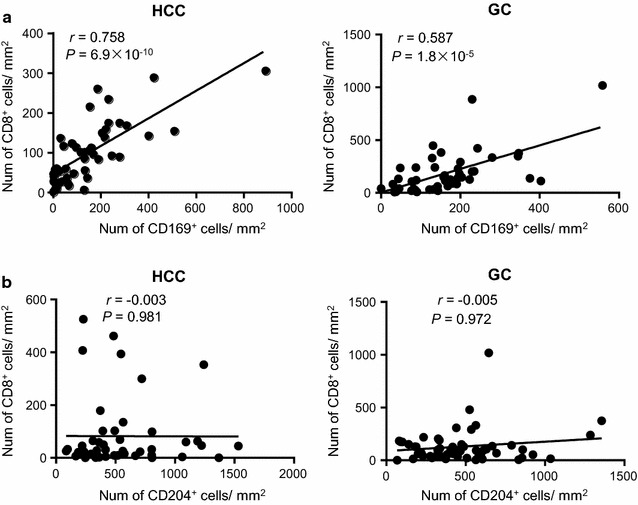
The density of CD169^+^ Mφs was positively associated with CD8^+^ T cells in both HCC and GC tissues. Immunohistochemical quantification showing the associations between the densities of CD169^+^ Mφs (**a**) or CD204^+^ Mφs (**b**) and those of CD8^+^ T cells in the intra-tumor regions of HCC and GC tissues. Correlations were performed by Spearman’s rank correlation coefficient test

We also tested the association of CD204^+^/CD169^+^ Mφ density with CD8^+^ T cell infiltration. As shown in Fig. 4, the density of CD169^+^ Mφ was positively correlated with CD8^+^ T cells infiltration in both HCC and GC (*P* < 0.0001), indicating the anti-tumor functions of these Mφs in both tumors. However, no association was found for CD204^+^ Mφ in either HCC or GC (*P* > 0.05). The associations between the CD204^+^/CD169^+^ Mφ density and clinicopathological variables have also been analyzed. The density of CD204^+^ cells was significantly correlated with tumor number, tumor size, TNM stage and histological grade (*P* = 0.006, *P* = 0.004, *P* = 0.004, and *P* = 0.002, respectively; Table 3) in HCC patients. No significant correlations were found between CD169^+^ cells density and clinicopathological variables in either HCC or GC.

**Table 3:** Association of Mφ with patients’ clinical characteristics

Characteristics	CD204^+^ Mφs^a^		*P* ^b^	CD169^+^ Mφs^a^		*P* ^b^
	Low	High		Low	High	
HCC patients						
Gender (male/female)	73/19	81/10	0.104	75/19	81/10	0.105
HBV infection (no/yes)	7/85	11/80	0.333	8/86	11/83	0.629
Alpha-fetoprotein, ng/mL (≤25/>25)	42/50	30/61	0.096	36/58	38/56	0.881
Child–Pugh class (A/B)	85/7	85/6	1.000	89/5	86/8	0.567
Tumor number (single/multiple)	78/14	61/30	*0.006*	69/25	75/19	0.389
Tumor size, cm (≤5/>5)	48/44	28/63	*0.004*	38/56	42/52	0.658
Vascular invasion (absent/present)	87/5	85/6	0.767	88/6	89/5	1.000
TNM stage (I/II + III)	72/20	53/38	*0.004*	63/31	67/27	0.636
Histological grade (I/II/III/other)	71/21	50/41	*0.002*	68/26	57/37	0.122
GC patients						
Gender (male/female)	45/21	51/14	0.237	45/21	50/16	0.439
Tumor size, cm (≤4/>4)	23/43	20/45	0.711	22/44	23/43	1.000
Tumor depth (pT1 + pT2 pT3/pT4)	20/46	21/44	0.852	22/44	24/42	0.855
Lymph node metastasis (pN0 + pN1/pN2 + pN3)	32/34	23/42	0.158	28/38	29/37	1.000
TNM stage (I + II vs. III + IV)	19/47	15/50	0.551	16/50	21/45	0.439
Histological grade (I + II/III/other)	19/42/5	14/45/4	0.637	22/37/6	13/49/4	0.112

*HCC* hepatocellular carcinoma, *GC* gastric cancer, *HBV* hepatitis B virus, *TNM* tumor-lymph node-metastasis

^a^Data were missing for these variables in some patients: CD204^+^ Mφs, n = 183 and CD169^+^ Mφs, n = 188 in HCC; CD204^+^ Mφs, n = 131 and CD169^+^ Mφs, n = 132 in HCC

^b^Italic values indicate significance of *p* value (*p* < 0.05)

### Prognostic power of the Mφ index in HCC and GC

Our findings indicated that the density of CD204^+^ and CD169^+^ Mφs represented a valuable independent factor to predict the prognosis of HCC. Therefore, we analyzed whether the combination of intra-tumor CD204^+^ and CD169^+^ Mφs (namely, the Mφ index) could represent a more powerful criterion for predicting patient prognoses.

In HCC, patients in the CD204^low^ and CD169^high^ group exhibited the best OS (5-year OS rate: 90.3%) compared with those in the CD204^low^ and CD169^low^ group (5-year OS rate: 64.2%, *P* = 0.019), CD204^high^ and CD169^high^ group (5-year OS rate: 63.0%, *P* = 0.01) and CD204^high^ and CD169^low^ group (5-year OS rate: 38.9%, *P* < 0.0001; Fig. 3c). A similar trend was found in GC patients, but did not reach statistical significance. In addition, we also analyzed the prognostic value of CD204^+^/CD169^+^ Mφs ratio, and found high CD204^+^/CD169^+^ Mφs ratio was correlated with poor survival in HCC patients (*P* < 0.001 Fig. 3d). In the multivariate Cox analysis, the Mφ index in HCC was also associated with OS in HCC, but not in GC (Additional file 1: Table S1).

## Discussion

Mφs form a major component of the inflammatory infiltrate in tumors, where they exhibit distinct phenotypes and diverse functions. In the present study, we investigated the distribution and composition of Mφ subpopulations in the NT and IT regions HCC and GC. Using CD204 and CD169 as subpopulation markers for Mφs, we found that most CD68^+^ Mφs were CD204^+^ and CD169^+^ cells in the NT region of HCC; however, the percentage of CD169^+^ Mφs deceased in the IT region. In contrast, CD68^+^ Mφs comprised a lower percentage of CD204^+^ than CD169^+^ subpopulations in the NT region, while more CD204^+^ but fewer CD169^+^ cells were in the IT region of GC. Moreover, the density of CD204^+^ Mφs was correlated with poor prognosis in HCC; however CD169^+^ Mφs are associated good survival in both HCC and GC.

In previous studies, various subpopulations of tumor-associated Mφs were identified; however, conflicting prognostic data was reported [31]. CD68, a glycoprotein predominantly resident in intracellular granules, is a fairly specific marker for pan-Mφs. In HCC, we and other groups have demonstrated that the number of CD68^+^ Mφs in tumor o was negatively correlated with patient prognosis [15, 16]. However, the data for GC is conflicting. For example, in some studies, CD68^+^ Mφs were correlated negatively with patient prognosis [32]; whereas, we and other groups have shown that GC patients with a high tumor-associated macrophage (TAM) count had better outcomes than those with a low TAM count [17, 33]. The discrepancies are probably a consequence of differences in the number, stage and size of tumors. In addition to these markers, there are also other phenotypes of Mφs, such as CD163^+^ and CD11c^+^ Mφs that exist in the different regions of tumors, which deserve further investigation [34, 35].

In addition to potentially representing a Mφ biomarker, CD204, a cell-surface glycoprotein that belongs to the scavenger receptors that has a pro-tumoral function during tumor progression [36], is associated with activation of Mφs toward an alternative or tumor-promoting and immunosuppressive phenotype. Accordingly, significant correlations between CD204 and negative outcomes have been reported across multiple tumor types [22–24]. CD169, also known as Siglec-1, belongs to the sialic-acid-binding immunoglobulin-like lectin family, which includes molecules that can mediate cell–cell interactions via glycan recognition [37]. The expression and function of CD169 on TAMs are poorly understood. Our recent study revealed the anti-tumor function of CD169^+^ Mφs in HCC [28]. In the present study, we confirmed the positive prognostic role of CD169^+^ Mφs in HCC. Moreover, we found that the number of CD169^+^ Mφs also correlated with good prognosis in GC patients. The function of CD169^+^ Mφs during GC progression deserves further investigation. Taken together, our results showed that the CD204^+^ and CD169^+^ Mφ subpopulations have diverse prognostic values during tumor progression.

Recent studies in mouse models revealed that Mφs can be generated from distinct sources in different organs, and the local environments might influence the function of Mφs [38, 39]. In this study, we found distinct composition patterns of these Mφ subpopulations within the NT and IT regions of HCC and GC, suggesting that environmental tissue factors in the gut and liver might contribute to the distinct developments of Mφs. We further found that the CD169^+^ Mφs density were correlated with more CD8^+^ T cells infiltration and good prognosis in both HCC and GC, indicating the anti-tumor functions of these Mφs in both tumors. These data suggested a similarity function but distribution differences for Mφ subpopulations in different tumors. The underlying mechanisms that regulate the infiltration and development of Mφ subpopulations, such as their epigenetic and transcriptional features which might be influenced by local environmental factors, deserve further investigations.

Based on the data that most cancers are populated by M2 Mφ, preclinical and clinical studies in several solid tumor types are designed using CSF-1R inhibitors or blocking monoclonal antibodies to reduce the presence of TAM [4, 40, 41]. However, due to the plasticity of Mφ, modulating M2 to M1 Mφs that could stimulate Th1-type cytotoxic T cells and other effector cells are emerged as an important strategy for immunotherapy of cancer [42]. Considering the importance of the protective function and prognostic role of CD169^+^ Mφ in both HCC and GC, it may be worth investigating whether the selective overexpression of CD169 might represent a novel therapeutic approach to reprogram the anti-tumor activities of Mφ.

## Conclusions

Our study demonstrated that CD204^+^ and CD169^+^ Mφ subpopulations display tissue-specific distributions and distinct composition patterns in different tissue micro-localizations, and have diverse prognostic values during tumor progression in HCC and GC. The results could help to reveal the possible therapeutic implications of Mφs and how to restore the anti-tumor properties of Mφs for immunotherapies.

## Additional files

**Additional file 1: Figure S1.** Coexistence of CD169, CD204 and CD68 in intra-tumor (IT) of HCC and GC tissues. **Figure S2**. Composition patterns of CD204^+^ Mφs and CD169^+^ Mφs subpopulations in CD68^+^ Mφs of HCC and GC non-tumor (NT) tissues. **Table S1**. Univariate and multivariate analyses of variables associated with overall survival.

**Additional file 2.** The original data of HCC cohort with clinicopathological variables.

**Additional file 3.** The original data of GC cohort with clinicopathological variables.

## Abbreviations

HCC: hepatocellular carcinoma
GC: gastric cancer
Mφ: macrophage
IHC: immunohistochemistry
IT: intra-tumor
NT: non-tumor
FFPE: formalin-fixed paraffin-embedded
OS: overall survival
