# Supplementary material for: Distinct patterns and prognostic values of tumor-infiltrating macrophages in hepatocellular carcinoma and gastric cancer
Source: J Transl Med. 2017 Feb 15;15:37. doi: 10.1186/s12967-017-1139-2 (PMC5312581; doi:10.1186/s12967-017-1139-2)
Supplement: Supplementary file 1 — Additional file 1: Figure S1. Coexistence of CD169, CD204 and CD68 in intra-tumor (IT) of HCC and GC tissues. Figure S2. Composition patterns of CD204+ Mφs and CD169+ Mφs subpopulations in CD68+ Mφs of HCC and GC non-tumor (NT) tissues. Table S1. Univariate and multivariate analyses of variables associated with overall survival. [file 12967_2017_1139_MOESM1_ESM.pdf]

Supplementary Figure S1

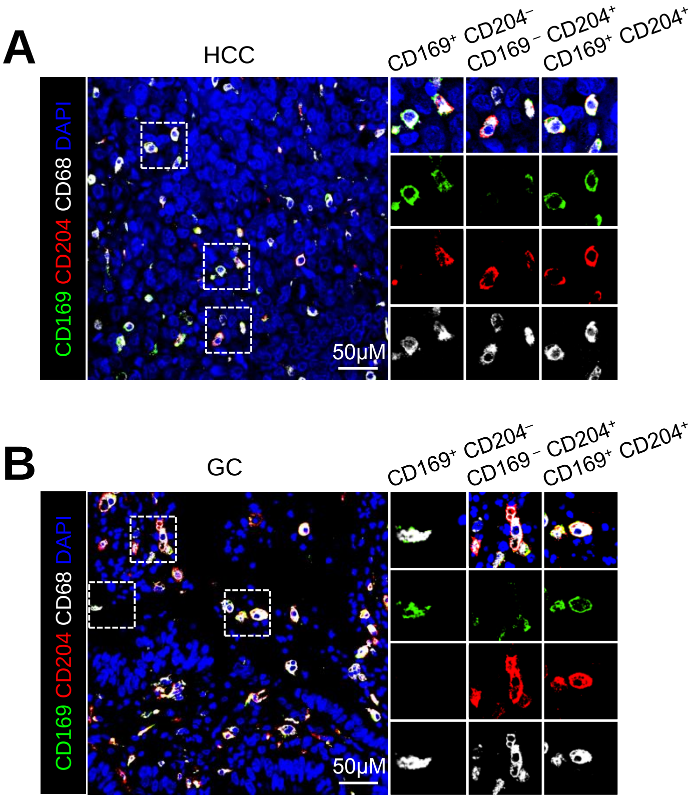

Supplementary Figure S1. Coexistence of CD169, CD204 and CD68 in intra-tumor (IT) of HCC and GC tissues. (A) Paraffin-embedded tissue sections (n = 5) were subjected to three-color immunofluorescence for CD169 (green), CD204 (red) and CD68 (gray) with DAPI counterstaining (blue) in HCC (A) and GC (B) tissues. Representative images of intra-tumor region were shown. Scale bar, 50 μm.

Supplementary Figure S2

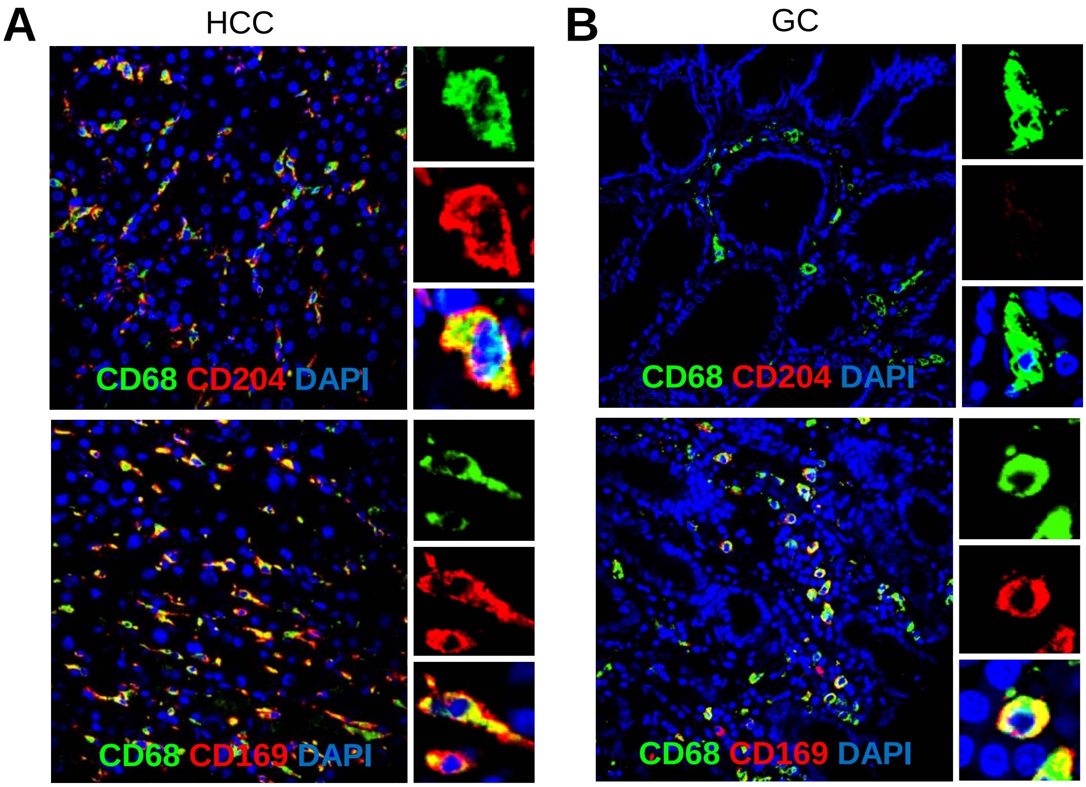

Supplementary Figure S2. Composition patterns of CD204⁺ Mφs and CD169⁺ Mφs subpopulations in CD68⁺ Mφs of HCC and GC non-tumor (NT) tissues. Paraffin-embedded tissue sections (n = 5) were subjected to two-color immunofluorescence for CD204 (red) or CD169 (red) and CD68 (green) with DAPI counterstaining (blue) in the NT regions of HCC (A) and GC (B) tissues.

Supplementary Table 1. Univariate and multivariate analyses of variables associated with overall survival[a]

Variables	Univariate			Multivariate		
	**HR**	**95% CI**	***P***	**HR**	**95% CI**	***P***
**HCC patients:**						
Gender (male / female)	1.333	0.747-2.377	0.331			
HBV infection (no / yes)	0.863	0.431-1.729	0.678			
Alpha-fetoprotein, ng/mL (≤25 / > 25)	1.023	1.003-1.043	**0.027**			NA
Child-Pugh class (A / B)	1.626	0.782-3.378	0.193			
Histological grage (I / II / III / other)	1.42	0.901-2.237	0.131			
Tumor number (single / multiple)	3.178	2.008-5.031	**< 0.0001**			NA
Tumor size, cm (≤5 / >5)	1.838	1.154-2.927	**0.01**			
Vascular invasion (absent / present)	3.832	1.825-8.046	**< 0.0001**	2.898	1.328-6.324	**0.008**
TNM stage (I vs II + III)	3.383	2.164-5.289	**< 0.0001**	2.851	1.781-4.465	**0.0002**
Mφ index						
CD204^{high} and CD169^{high}			NA			NA
CD204^{low} and CD169^{low}	0.924	0.530-1.609	0.779	1.031	0.590-1.802	0.915
CD204^{high} and CD169^{low}	2.115	1.197-3.736	**0.01**	2.113	1.189-3.754	**0.011**
CD204^{low} and CD169^{high}	0.336	0.138-8.17	**0.016**	0.371	0.152-0.904	**0.029**
**GC patients:**						
Gender (male / female)	1.189	0.702-2.013	0.52			
Tumor size, cm (≤4 / >4)	1.655	0.951-2.881	0.075			
Tumor depth (pT1+pT2+pT3 / pT4)	1.251	0.744-2.105	0.398			
Lymph node metastasis (pN0+pN1 / pN2+pN3	1.999	1.192-3.353	**0.009**	1.999	1.192-3.353	**0.009**
TNM stage (I+ II vs III + IV)	2.031	1.103-3.738	**0.023**			NA
Histological grade (I / II / III / other)	1.18	0.772-1.804	0.445			
Mφ index						
CD204^{high} and CD169^{high}			NA			
CD204^{low} and CD169^{low}	1.398	0.739-2.645	0.303			
CD204^{high} and CD169^{low}	1.273	0.613-2.645	0.517			
CD204^{low} and CD169^{high}	0.708	0.308-1.629	0.417			

Abbreviations: HCC, hepatocellular carcinoma; GC, gastric cancer; HBV, hepatitis B virus; TNM, tumor-lymph node-metastasis; Mφ, macrophage; CI, confidence interval; NA, not applicable.

[a]Cox proportional hazards regression model; variables that were associated with overall survival in the univariate analysis were adopted as covariates in the multivariate analysis and entered into the equation by using the forward likelihood ratio method.
